# Unmasking non-malarial mosquito-borne infections among febrile children in malaria-endemic regions of western Kenya

**DOI:** 10.1038/s41598-026-47471-0

**Published:** 2026-04-07

**Authors:** Jack Ogony, Simon Karanja, George Ayodo, Ivy Akinyi, Daniel Onguru, Charles Angira, Diana Menya, Judith Mangeni

**Affiliations:** 1 Department of Environmental Health and Disease Control, Jomo Kenyatta University of Agriculture and Technology, Nairobi, Kenya; 2 School of Health Sciences, Jaramogi Oginga Odinga University of Science and Technology, Kisumu, Kenya; 3 Department of Epidemiology and Biomedical Statistics, Moi University, Eldoret, Kenya

**Keywords:** Arboviruses, Non-malarial, Febrile illness, Climate change, Resistance, Dengue fever, Diseases, Health care, Medical research, Microbiology

## Abstract

Almost half of under-five children in health facilities are febrile in sub-Saharan Africa. The non-specific clinical symptoms coupled with; limited diagnostics capacities, overlapping endemicity and the surveillance gaps complicates accurate cause identification of an acute febrile illnesses in resource limited settings. This challenge leads to misdiagnosis, overtreatment, and delays in appropriate management, increasing morbidity and mortality. Health systems are often overloaded, with providers attributing fevers to the most common pathogen, while other emerging infections are the cause. This study unpacked the febrile illness by testing for dengue fever in parallel to malaria. Febrile ill children below 5 years seeking health services public health facilities in Busia and Kisumu Counties were screened using an approved malaria and dengue fever rapid test kits at the outpatient department. Those who screened positive were recruited into the study. A total of 1004 children were screened, 380 met the recruitment criteria. 215 (21.4%) tested positive for *P. falciparum* alone, 90 (8.9%) tested positive for dengue fever alone while 75 (7.5%) had co-infections. Busia had the highest *P. falciparum*-only infection (23.4%) while Kisumu had the highest dengue-only infections (12.6%). Dengue fever is a re-emerging neglected tropical, climate change driven disease in malaria endemic regions. Other than creating awareness to build capacity for diagnosis, this study unmasked and confirmed dengue as a major contributor to the non malarial febrile illnesses among children. There is need to revise the screening algorithm for febrile patients to improve arboviral surveillance.

## Introduction

Mosquito-borne pathogens pose an increasingly serious threat to global health, especially in tropical and subtropical regions because of their recurring outbreaks^1^. World Health Assembly (WHA), the decision-making body of the World Health Organization (WHO), endorsed aims to reduce malaria burdens a further 90% by 2030, with the possibility of malaria eradication following a significant reductions in malaria morbidity and mortality^2,3^. The new area of research, however is focused on identifying etiologies of non-malaria, acute febrile illness (AFI) as most of the cases often have similar non-specific clinical presentations^5^. Despite the control efforts, malaria is still a serious, a life-threatening infectious disease affecting humans and caused by five different species of protozoan parasite and transmitted by the bite of an infected female anopheles mosquito^6^. Among the five species, *Plasmodium falciparum (P. falciparum)* still remains an overwhelming threat in Africa, harbouring approximately 90% of global cases^7^. Malaria manifests as a febrile illness and is fatal if unrecognized in young children^8^. Despite the apparent progress in malaria control, including the current routine RTS, S malaria immunizations program targeting the high-transmission counties in Lake Region of western Kenya^9^, there were still more cases and deaths in sub-Saharan African countries among children under five years of age according to WHO^10^. Under 5 years children remain vulnerable as malaria is still the leading cause of morbidity and mortality in Western Kenya, particularly in the Lake Victoria endemic region^11^.

Studies have also shown that disease burden could shift from malaria to arboviruses especially in sub-Saharan Africa due to the impacts of climate change^12^. Climate change intensifies the spread of these diseases via various mechanisms; expanded geographic range, accelerating vector and pathogen development, increased biting rates, altering the breeding habitats and through disruption of the ecosystems^13^. Dengue is transmitted predominantly by two mosquito vectors female mosquitoes species; *Aedes aegypti* and *Aedes albopictus* and the disease is caused by any of the 4 serotypes of dengue viruses (DENV);1, 2,3 and 4^14,16^. Acquiring any of these serotypes confer lifelong immunity to that specific serotype, but not a protection against the others, so individuals may be affected up to four times over their lifetime^17^. About 40% of the global population are at risk of dengue acquisition and up to 400 million people are infected with DENV annually, 50–100 million people get sick, and more than 20,000 people die from severe dengue^18^. Globally, dengue cases have increased > 10-fold between 2000 and 2019 (500,000 to 5.2 million), an indication of epidemiological expanding of the disease^19^. The disease can easily be misdiagnosed due to its non-specific features. Its public health impact in Kenya has continued to rise sharply, with successive recent outbreaks resulting in hundreds of thousands of human cases and multiple fatalities^20^. Moreover, in recent decades, an increase in the coinfection and circulation of cases caused by the dengue virus and *P. falciparum* in the same region have been reported in malaria endemic areas^21,22^. Malaria and dengue fever are the major cause of febrile illness among the patients seeking healthcare services^23^. Currently, there is neither a vaccine nor any specific therapy for severe dengue infection, the progress on both fronts have met incomplete understanding of disease pathogenesis^24^.

Identifying etiologies of AFI is challenging due to non-specific presentation and limited availability of diagnostics especially in malaria endemic settings. Febrile illness (defined as temperature > 38 °C^25^, without localizing features is the most common reasons for seeking healthcare services in sub Saharan Africa^26^. Patients presenting with fever in endemic areas, clinical diagnosis is always difficult and malaria remains the default diagnosis. In such areas, dispensing of antimalarial may seldom follow the WHO guidelines of confirming a malaria infection in febrile children through a diagnostic test before giving antimalarial treatment^27,28^. This has created a diagnostic dogma for health workers at the facility or community level, by having a large group of patients with “Non-malarial febrile illnesses (NMFI)” and with few or no diagnostic options available to guide the subsequent management of these febrile cases.

Non-malarial febrile illnesses are infectious diseases affecting patients who show signs of indistinguishable fever thus necessitate use of malaria rapid diagnostic tests (mRDTs) in resource limited setting, however, these tests turn out to be negative for malaria^29^. Laboratory assays for many febrile diseases are often complex, costly, and not widely available in areas where epidemiologic information on the etiology of febrile illness is sparse^30^. The NMFIs account for about half of all fever presenting morbidities among under-five children in sub-Saharan Africa and mostly caused by bacteria and viruses^26,31^. This implies the need for ‘test before treat’ as opposed to presumptive treatment of fevers as malaria, to avoid unwanted consequences as many studies have reported unnecessary prescription of antimalarial and antibiotic^32^.

Malaria-oriented health systems have ignored many other infectious diseases, including arboviral diseases such as dengue fever which are emerging yet health threat in Africa linked with the rising climate change^33^. The increased use of mRDTs has shown that many individuals with suspected malaria are not infected with *P. falciparum*. Although the incidence of *P. falciparum* malaria is falling, transmission is heterogeneous between regions, with higher incidence in western Kenya^34^. The most recognized cause of febrile illnesses has gradually been shifting from malaria to other infectious diseases^35^. A major clinical question paradoxically therefore arises as malaria diagnosis improves: what are the main diagnoses among febrile children without malaria and how should these individuals be managed? The treatment administered to non-malaria patients in all public health facilities is left to the discretion of the healthcare workers. However, few diagnostic facilities or data are available to identify the non-malarial mosquito borne disease responsible for their febrile state to guide these decisions.

In Kenya, the community health promoters (CHP) are trained to undertake mRDTs and to give antimalarial drugs to patients who test positive. When the results are negative with a febrile child, the CHP are left in dilemma, thus information is needed to develop algorithms to manage febrile patients with no malaria. To be effective, such algorithms should take into account heterogeneity in incidence and epidemiology of infectious disease across a country. Currently, there is a paucity of information regarding testing of dengue fever and its distribution in Kenya. Furthermore, there is currently no consensus on how to report NMFI etiology results, making it difficult to view distribution across time and space. We therefore prospectively introduced a dengue fever rapid testing parallel to the malaria rapid tests to unpack acute febrile fevers transmitted by mosquito other than *P. falciparum*, the most dominant malaria parasite in western Kenya.

## Materials and methods

### Study site

**Fig. 1:**
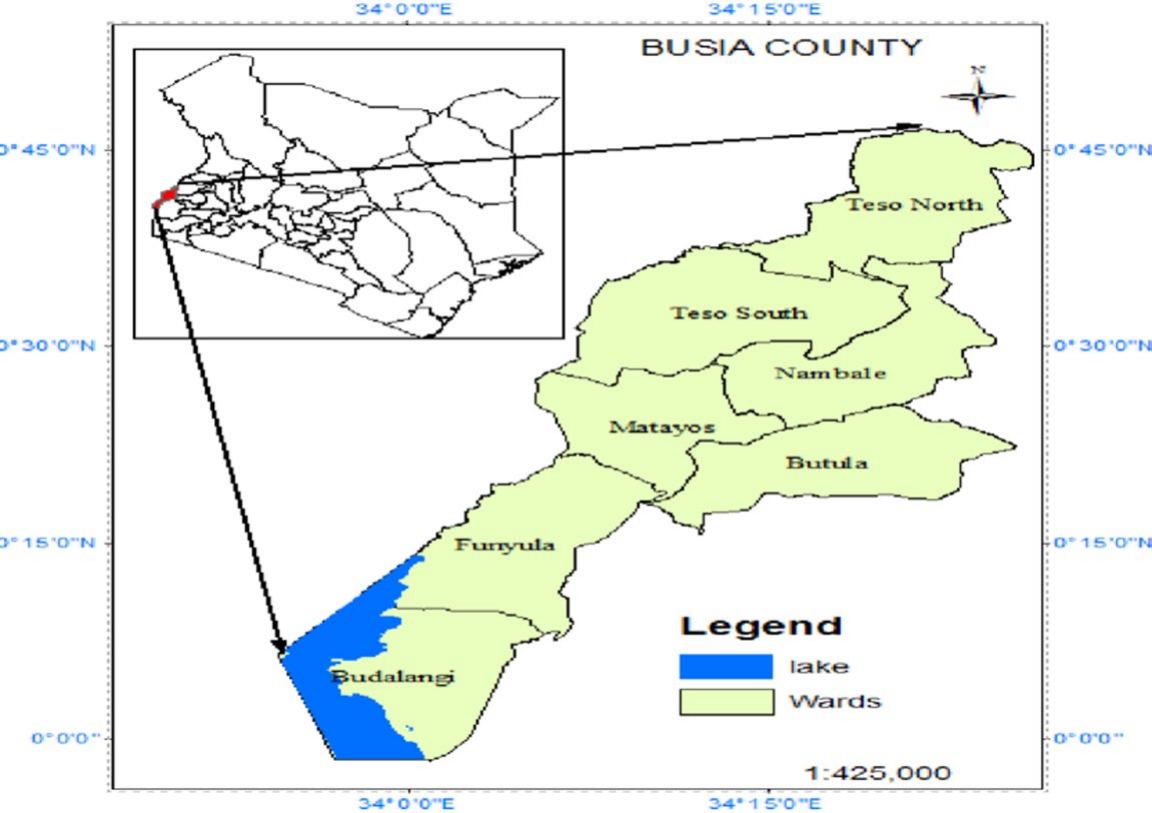
Map showing Sub Counties of Busia (modified from journal article) (https://www.researchgate.net/figure/Map-of-Busia-County-Kenya_fig1_385458440).

The study was done in Kisumu City (an urban setting) and Busia County (a peri-urban area) in western Kenya. This is a malaria endemic region and also experience mosquito vector invasion due to climate-related events^36^. The specific sites were Bunyala Sub-County in Busia County and Kisumu Central Sub-County in Kisumu City. Busia borders Lake Victoria to the South West, the Republic of Uganda to the West, Siaya County to the southeast, and Bungoma County and Kakamega County to the east. The county is composed of six sub-counties, and has a population of 893,681, according to the recent National Census^37^. The county’s climatic conditions are greatly affected by Lake Victoria (Fig. 1).

**Fig. 2:**
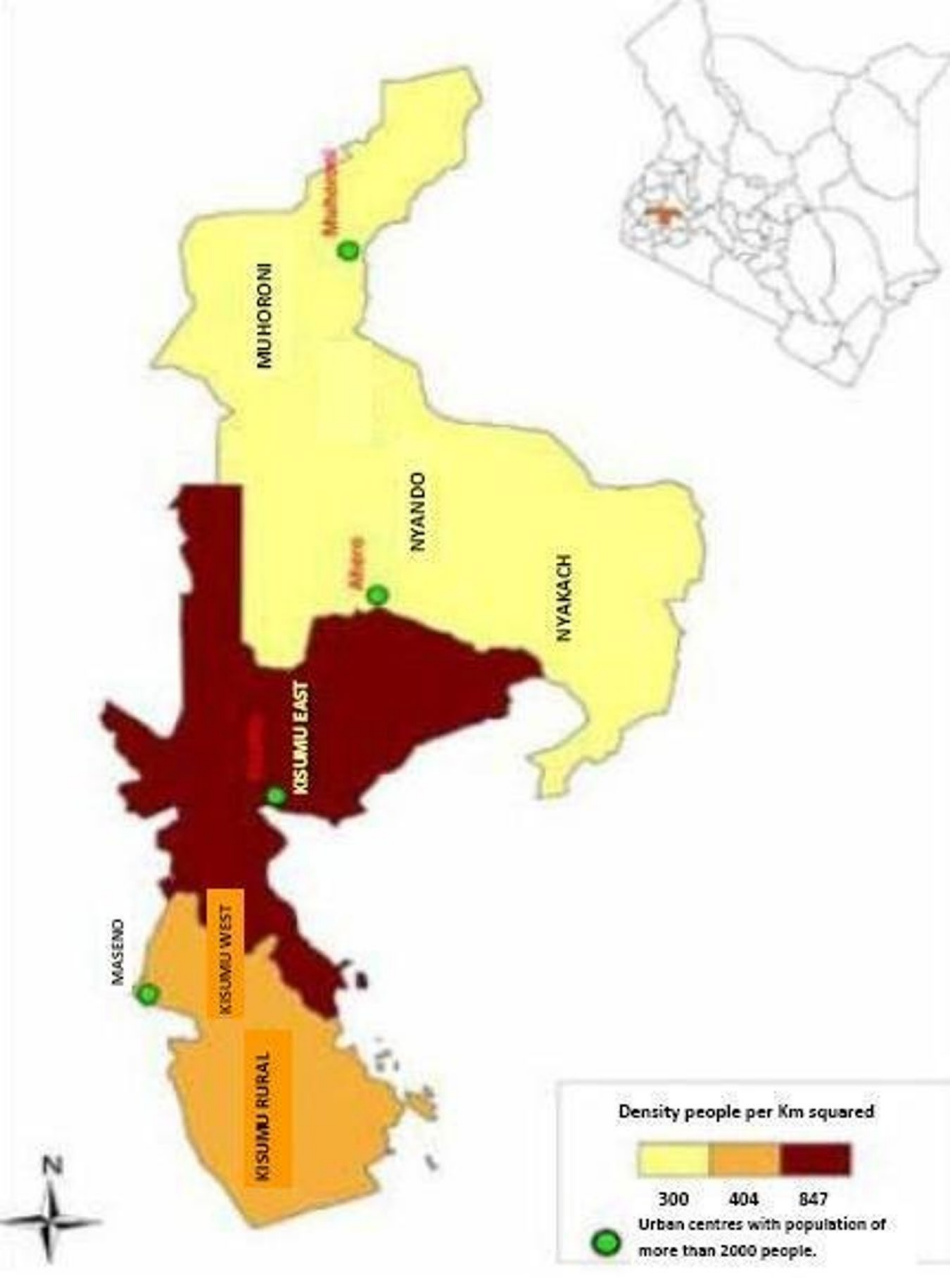
Map showing sub-counties of Kisumu County (modified from journal article) (https://www.researchgate.net/profile/AgeviHumphrey/publication/310753270/figure/fig8/AS:784912667922432@1564149171770/Map-showing-sub-counties-of-Kisumu-county_W640.jpg).

Kisumu is the third-largest city in Kenya located in the Lake Victoria area. It borders Homa Bay County to the South, Nandi County to the North East, Kericho County to the East, Vihiga County to the North West and Siaya County to the West. It is the East African commercial port and transportation Centre of western Kenya. According to 2019 National Census, Kisumu County has a population of 1,155,574^37^. The climate is consistently warm and humid, with relatively high humidity levels throughout the year. The average annual temperature of Kisumu is around 22.9 °C. Kisumu is susceptible to flooding and extreme temperatures, the shoreline floods are a concern due to the rise in Lake Victoria’s water level, while riverine floods occur along the rivers flowing into the lake. Kisumu central sub county is the urban center surrounded by Kisumu East, Kisumu West, Seme, Nyando, Muhoroni, and Nyakach sub-counties (Fig. 2).

### Study design

This was a hospital based, prospective study among febrile children below 5 years seeking health services at the public health facilities.

### Data collection

All the research assistants were trained on the tools and diagnostic procedures. The tools were also pre-tested in a different health facility before actual data collection to ensure validity and reliability.

### Inclusion criteria

All febrile children who screened positive for *P. falciparum* and or dengue fever were recruited. The parents/guardian must have voluntarily agreed to sign informed consent form.

### Exclusion criteria

Children who were severely ill or tested negative for malaria or dengue fever or child in care were not recruited.

## Specimen collection and laboratory diagnostics

Whole blood samples were collected by finger prick done by a trained study clinician. Malaria and dengue RDTs were then immediately applied, as explained below;Dengue Virus Screening: OnSite^®^ CTK Biotech, Inc, 10110 Mesa Rim Road San Diego CA 92121, USA Duo Dengue Ag-IgG/IgM Rapid Diagnostic Kit, Batch 271158, Expiry Date 30/06/2025 was used to screen DENV infections among the children^38^. This is an antigen-antibody dual detection test, noninvasive diagnostic method which can be performed in 20–25 min even in a resource limited setting.

### Test principle

This is a lateral flow immunoassay for the simultaneous detection and differentiation of IgG anti-dengue virus, IgM anti-dengue virus and dengue NS1 antigen (DEN1, 2, 3, 4) in human serum, plasma or whole blood. The kit utilizes recombinant chimeric dengue virus and able to detects all four dengue serotypes. The test strip consists of; (1) a colored conjugate pad containing recombinant dengue envelope antigens conjugated with colloidal gold (dengue Ag conjugates) and a control antibody conjugated with colloidal gold, (2) a nitrocellulose membrane strip containing two test lines (G and M lines) and a control line (C line). The G line is pre-coated with antibodies for the detection of anti-dengue virus IgG, the M line is pre-coated with antibodies for the detection of anti-dengue virus IgM, and the C line is precoated with a control line antibody^39^.

### Test procedure

The test device was placed on clean, flat surface and labeled with the participant’s specimen’s ID. A high quality finger stick was aseptically performed by a trained and qualified laboratory officer. About 10 µL of whole blood was collected using capillary tube provided in the kit and dispense into the specimen well. Next, four drops of assay diluent were added into the same specimen well. The results were read at 20 min. The validity of the test was checked by the appearance of a control line on each strip. The used device was then discarded according to the facility waste management policy after interpreting and documenting the results.

### Result interpretation

Dengue Ag positive result suggested an active infection, IgM detection indicated recent infection; IgG detection indicated recent or previous infections. Dengue IgG and IgM positive result suggested late primary or early secondary infection. The kit has a shelf life of 24 months at a storage temperature of 2–30 °C. according to the kit insert, Duo Dengue Ag-IgG/IgM Rapid kit has Sensitivity of 97.3% (95% CI 85.8–99.9%), Specificity of 99.3% (95% CI 97.5–99.9%) and an overall Agreement of 99.1% (95% CI 97.3–99.7%) with no Cross-Reactivity^40^.

*P. falciparum* Screening : Since *P. falciparum* is the overwhelmingly dominant malaria parasite in this region, CareStart™ Malaria *Pf (HRP2) Ag* RDT, Access Bio, Inc 65 Clyde Rd., Suite A, Somerset, NJ 08873, USA (Multi Kit with capped lancet and inverted cup specimen transfer device) an MoH approved mRDT was used to perform the malaria testing^41^. The RDT is inexpensive, stable at high temperatures (40 °C) and mostly used in health units. Each kit has 25 chromatographic test strips fixed in a cassette, lysis buffer, a pack of 25 lancets and disposable alcohol swabs saturated with 70% isopropyl for disinfection^42^.

### Test principle

The kit is a lateral flow immuno-chromatographic antigen-detection RDT, which relies on the capture of dye-labeled antibodies to produce a visible band on a strip of nitro-cellulose, often encased in plastic housing, referred to as cassettes. With malaria RDTs, the dye-labeled antibody first binds to a parasite antigen, and the resultant complex is captured on the strip by a band of bound antibody, forming a visible test (T) line in the results window. A control (C) line gives information on the integrity of the antibody-dye conjugate but does not confirm the ability to detect parasite antigen. This test utilizes a pair of antibodies to detect *P. falciparum histidine-rich protein II (pHRP-II)* only hence not able to detect non-Pf (*P. vivax*,* P. ovale* and *P. malariae*) infections.

### Test procedure

About 5 µL of whole blood from a finger prick was applied to the test device’s sample well using a provided sample dropper. The test was timed, read at 20 min and documented.

### Result interpretation

A presence of T line and C line suggested a valid *P. falciparum* infection. Presence of C line with no test line suggested a valid *P. falciparum* negative result while absence of control C with or without test T suggested an invalid test, and must be repeated before declaring the result. This device has sensitivity of is 99.4%, specificity of 98%, positive predictive value of 94.4% and negative predictive value of 99.8%^43^.

### Study limitation

Due to resource limitations and the study setting, the conclusion were only based on the sensitivity and specificity of both *P. falciparum* and dengue fever specific RDTs. Other superior tests like PCR were not used to detect other malaria species other than *P. falciparum (P. vivax/P. malariae/P. ovale sp.).* The study site is however a *P. falciparum* dominant, responsible for nearly 100% of cases.

### Ethics statement

The study procedures were performed in accordance with the ethical standards as stipulated in the 1964 Helsinki Declaration and its later amendments or comparable ethical standards. The research was approved by the Institutional Scientific Ethics Review Committee (ISERC) of Jaramogi Oginga Odinga Teaching and Referral Hospital ref no. ISEC/JOOTRH/752/23) and licensed by the National Commission for Science, Technology, and Innovation (NACOSTI), license No. NACOSTI/P/23/32018. It was also authorized by both county governments of Kisumu (ref: GN133VOL.XV/250) and Busia (ref: CG/BSA/H/ADM/1/56/IX).

The participants were only recruited after going through the informed consent and signed a consent form. The consenting session included explanations of; the purpose of the study, the study activities, and the risks associated with the study, the study procedures highlighting the participants’ right to discontinue participation at any time without penalty or loss of benefits. The study population directly benefitted by freely knowing their malaria or dengue sero status which was sponsored by the study. All the participants who tested positive either for malaria or dengue fever were treated as per the national MoH guidelines at the same health facility based on the facility protocol. At the same time, the febrile participants who tested negative for either malaria or dengue virus were equally managed based on MoH protocol such as assessment for danger signs and tailored antibiotic treatment based on clinical guidelines.

### Data management and analysis

Data was automatically transmitted and stored in a study password-protected computer. Data cleaning was done and analyzed using StataCorp 15. The Chi-square and Fisher exact test was used to compare categorical variables. Descriptive statistics included sociodemographic characteristics of the participants. Inferential analysis such as logistic regression analysis was used to test significant association between variables (*P* ≤ 0.05). Odds ratios were calculated with 95% confidence intervals (*P* < 0.05). Statistical data on the prevalence of dengue, malaria and dengue–malaria co-infection was used for analysis of the correlation between prevalence and the study sites.

## Results

**Table 1:** Sociodemographic characteristics of the study participants.

Variable	Overall *N*, = 380	Busia, *n* = 190	Kisumu, *n* = 190	*p*-value
Child’s gender				0.039
Female	174 (46.0%)	97 (51.0%)	77 (41.0%)	
Male	206 (54.0%)	93 (49.0%)	113 (59.0%)	
Child school going?				0.4
Not started school	228 (60.0%)	110 (58.0%)	118 (62.0%)	
Started school	152 (40%)	80 (42.0%)	72 (38.0%)	
Parent’s marital status				0.2
Married	306 (80.5%)	158 (83.0%)	148 (78.0%)	
Single	74(19.5%)	32 (17.0%)	42 (22.0%)	
Parent’s highest education				< 0.001
College/vocational training	73 (19.5%)	24 (13.0%)	49 (26.0%)	
Primary and below	138 (36.0%)	84 (44.0%)	54 (28.0%)	
Secondary level	169 (44.5%)	82 (43.0%)	87 (46.0%)	
Parent’s source of income (salaried/business)				< 0.001
No	188 (49.5%)	117 (62.0%)	71 (37.0%)	
Yes	192 (50.5%)	73 (38.0%)	119 (63.0%)	
Peasant farmer				< 0.001
No	294 (77.5%)	114 (60.0%)	180 (95.0%)	
Yes	86 (22.5%)	76 (40.0%)	10 (5.0%)	

The mean age of the children was 3.07(1.60, 4.60) years. On average, older children (3.60 (2.40, 4.55)) had more co-infections while younger ones (2.60 (1.63, 3.60)) had dengue fever only infections. Out of the 380 children recruited, 174(46.0%) were female where 97 (51.0%) of them being residents of Busia. Majority of the males (113 (59.0%)) were residents of Kisumu city. At the time of this research, most of the children (228 (60.0%)) had not started going to school, among the once already school-going, 80 (42.0%) and 72 (38%) were residents of Busia and Kisumu respectively. The parents/ guardians who at least reached primary level of education were 138 (36.0%) with majority them (84 (44.0%)) in Busia County. Majority of those who were college or vocational training level graduates were the lowest (73 (19.5%))(Table 1).

**Table 2:** Diagnosis burden among the febrile Children per study site.

County	Total screened	*P. falciparum*	Dengue fever	Concurrent infections
Busia	505 (50.3%)	118 (23.4%)	27 (5.3%)	45 (8.9%)
Kisumu	499 (49.7%)	97 (19.4%)	63 (12.6%)	30 (6.0%)
Overall	1004	215 (21.4%)	90 (8.9%)	75 (7.5%)

**Fig. 3:**
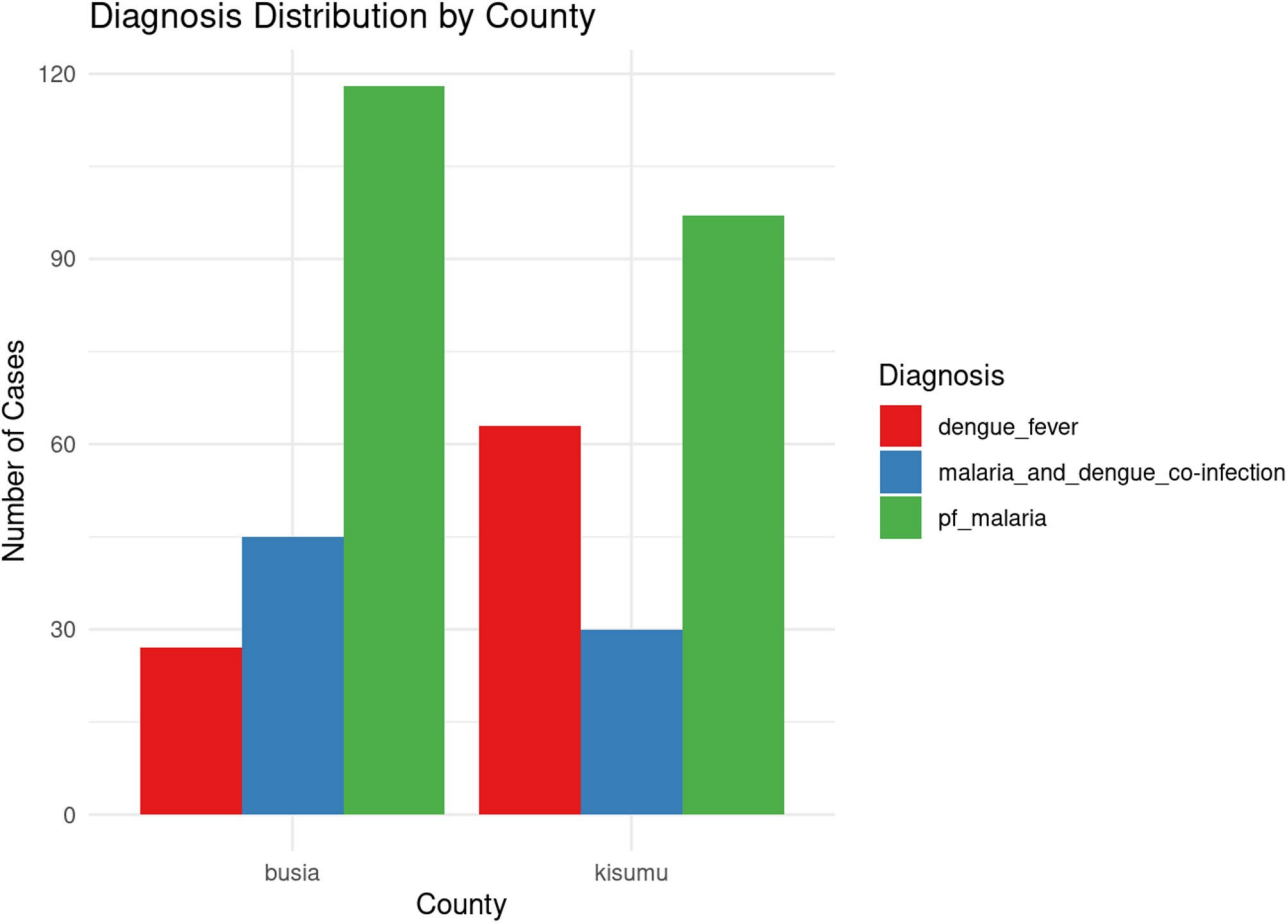
Distribution of mosquito-borne infections in study sites.

Out of the total 1004 febrile children screened, 215 (21.4%) had *P. falciparum*, 90 (8.9%) had dengue fever infection while the remaining 75(7.5%) had coinfections. Busia had more cases of *P. falciparum* compared to Kisumu (23.4% versus 19.4%, *p* < 0.005) whereas Kisumu had more cases of dengue fever than Busia (12.6% versus 5.3%, *p* < 0.005). Similarly, more coinfections were recorded in Busia than Kisumu (8.9% versus 6.0%, *p* = 0.081) (Table 2, Fig. 3).

The distribution of diagnoses across the two counties revealed notable differences in the occurrence of dengue fever, malaria, and co-infection. In Busia, *P. falciparum* was the most common diagnosis, accounting for about 62.1% of the cases, followed by co-infection (23.7%), and dengue fever alone (14.2%). Kisumu site, on the other hand had a higher proportion of dengue fever cases (33.2%) with *P. falciparum* comprising 51.1% and co-infection accounting for 15.8%. These data suggest a relatively higher occurrence of dengue fever in Kisumu, while Busia experiences more co-infections cases. Malaria infection is still leading in these regions. However there is a second and competing infections of dengue alone or co-infections. In Busia, 45 participants had concurrent infections, while in Kisumu, 30 individuals were co-infected (Fig. 3).

A chi-square test of independence between the counties and diagnosis was statistically significant (X^2^ = 19.45, *p* < 0.001), indicating that the distribution of these diagnoses is not random but rather associated with the location. There was negative correlation between having malaria infection and having dengue fever (*r* = − 0.636), implying that the two infections tended not to occur independently.

## Discussion

The transmission efficiency of mosquito-borne disease is dependent on critical components of vectorial capacity such as mosquito density, survival and human feeding preference^44^. Mosquito abundance increases the likelihood of vector-human contact and is an important parameter in climate-based models to predict disease dynamics. Among the mosquito-borne illnesses causing concern globally, *P. falciparum* and dengue fever are experiencing a dramatic increase in indicated by this study. Studies have shown that climate change has considerably accelerated the spread of vectors throughout tropical and subtropical regions including new countries thus elevating transmissions^15^. This recent study further revealed an emergence of dengue fever in Kisumu City, an area historically known for malaria as the main cause of febrile especially in children^45^. This finding coincides with a study done by Kamau et al., (2023) which proved that *Aedes aegypti*, (the vector) abundance, survival, human-blood feeding was higher in the same locality^19^. Another study by Mulwa et al., (2025) showed the same vector (*Aedes aegypti aegypti*) has undergone a rapid geographic range expansion and is now common throughout Kenya, a vector historically confined to coastal regions^15^. Since the first recorded case in Africa in 1779, there have been a series of outbreaks of all the four DENV serotypes across the continent of Africa, including Kenya^18^. According to Näslund et al., (2021), the prevalence of dengue may be more pronounced in Africa than what research currently suggests since the dissemination of the main vectors, *Aedes aegypti* and *Aedes albopictus*, is substantial across the continent^46^. These vectors exist widely and may be under recognized due to the vector control strategies that are conventionally focused on *Anopheles* species^46,47^. A study done by Futami et al., (2020) in Kenya concluded that the relatively, cases of dengue were abundant in urbanized areas, consistent with known ecology^48^. Our study finding is in agreement with this study, there were more cases of dengue in Kisumu which is more urbanized than Busia, a peri-urban (both urban and rural experience-like setting). Dengue can easily be misdiagnosed due to its non-specific features asymptomatic nature of the patients, this complicates public surveillance, resulting in missed and yet increased transmission^49,50^. Sahu et al. (2023) further concluded that accurate prediction of dengue in the context of climate change is crucial for public health experts for timely prevention^50^.

Studies conducted by Bangoura et al., (2024) showed seroprevalence rates among non-febrile persons and patients with fever at 22% each^51^. Our current study, however among febrile children showed a seroprevalence of 8.9%. Coinfections is another complication which have been reported in countries with overlapping prevalence of disease. Gebremariam et al., (2023), illustrated that there is an increasing prevalence of malaria and acute dengue virus coinfection in Africa^52^. Multiple co-infections with DENV and *Plasmodium* (mostly *vivax*) have also been recorded in India, a country with well-recognized dengue and concurrent malaria activity^53^. This recent study however identified coexistence of dengue and *P. falciparum*, the most prevalent among the *Plasmodium* spp in the study area^54^. Other case studies among febrile patients have also revealed DENV and *Plasmodium* co-infection as associated with more severe symptoms than having either infection alone, including more hemorrhagic manifestations, jaundice, and kidney dysfunction^55^. The recent study did however, not include the disease severity outcomes.

## Conclusion

Climate change is exacerbating arboviral disease driven febrile illnesses. The transmission rates and the geographic ranges of these disease vectors has extended to newer regions. The causes of febrile illness is gradually shifting from malaria to arboviral disease-cause. This phenomenon could lead to increased; presumptive treatment, abuse of ACTs, increased drug resistance, resulting to morbidities and mortalities in the regions otherwise known as malaria oriented. Dengue fever is a re-emerging neglected disease responsible for the NMFI in western Kenya. The infrastructural gap such as unavailability of diagnosis platforms in regions traditionally known for malaria lead to complex management of febrile children. This study has unmasked and established dengue fever as a major contributor to NMFI in malaria endemic regions yet underdiagnosed. Introduction of a laboratory based dengue fever diagnosis can improve diagnostic precision and timely care of the febrile cases. The study has created awareness on the need to build capacity diagnosis by introducing a parallel test to augment the existing malaria testing. Finally, there is a need for a multicenter research to explore the serodistribution of the 4 DENV serotypes and to understand their clinical spectrum in the region and possibility of dengue virus coinfections with other *plasmodium* species infections other than *P. falciparum*.

## Data Availability

All data generated or analyzed during this study are included in this published article.
